# Relative survival following hemi-and total hip arthroplasty for hip fractures in Sweden

**DOI:** 10.1186/s12891-018-2321-2

**Published:** 2018-11-23

**Authors:** Szilard Nemes, Dennis Lind, Peter Cnudde, Erik Bülow, Ola Rolfson, Cecilia Rogmark

**Affiliations:** 1 Swedish Hip Arthroplasty Register, Centre of Registers Västra Götaland, Gothenburg, Sweden; 2 Department of Orthopedics, Institute of Clinical Sciences, Sahlgrenska Academy, University of Gothenburg, Gothenburg, Sweden; 3 Department of Orthopedics, Lund University, Skane University Hospital, Malmö, Sweden; 4 Department of Orthopedics, Hywel Dda University Healthboard, Prince Philip Hospital, Bryngwyn Mawr, Llanelli, UK

**Keywords:** Hip fracture, Excess mortality, Relative survival, Hip arthroplasty

## Abstract

**Background:**

Hip fractures are a common problem in the ageing population. Hip arthroplasty is the common treatment option for displaced intracapsular neck of femur fractures. Even though hip replacements are successful in restoring mobility, reducing pain and diminishing loss of health-related quality of life, the potential impact of a hip fracture on life expectancy as well as the postoperative mortality need consideration. The purpose of this study was to describe the mid-term relative survival rate for a cohort of Swedish patients whom underwent total- or hemiarthroplasty surgery following hip fracture. We also explored whether the survival rate is prosthesis-type specific and influenced by comorbidities, sex, socioeconomic and surgical factors.

**Methods:**

Using prospectively collected information of the *Swedish Hip Arthroplasty Register*-linked database we identified 43,891 patients operated between 2005 and 2012. Patient- and surgery-specific data in combination with socio-economic data were available for this analysis. We studied relative survival rate and used multivariable modelling with Cox Proportional Hazards Model in Transformed Time.

**Results:**

Compared to the Swedish general population the baseline excess hazard was very high in the first half year after the operation, thereafter the excess hazard decreased but remained non-negligible through the 8 years’ follow-up period. The mortality rate of males was higher compared to women. Higher Elixhauser comorbidity index (ECI) was associated with worsening survival. However, patients who had ECI = 0 had higher mortality than patients with ECI =1 the first 420 days post fracture. Patients with a hemiarthroplasty had a worse survival than patients with a total hip arthroplasty. Of the hospital types considered university hospitals had lower survival rate. Younger patients had a greater loss of expected life span than patients who suffer hip fracture in their more advanced ages.

**Conclusions:**

Swedish hip fracture patients who undergo arthroplasty surgery had a high excess hazard of dying in the first half year following surgery, and this excess hazard never subsided to negligible levels at least up to 8 years after surgery. Interestingly having no prior record of illnesses worsened the initial mortality. Men living alone had the highest long-term excess mortality.

## Background

From the onset of age 60 the residual life time risk for hip fracture for men and women is estimated to be around 5 and 10%, respectively [1]. The number of hip fractures are bound to increase as the population becomes older [2]. It is well-known that hip fracture patients have a high risk of dying, but the literature is not concordant on how large the excess mortality is, compared to the general population of the same age, on how long time the excess risk prevails or if there are differences between the sexes [3, 4]. In recent years, there has been an improvement in the immediate care of the hip fracture patient, with shorter time to surgery and mobilisation within the first postoperative day. There is however conflicting evidence as to whether this actually has led to reduced mortality. Pedersen et al. [5] found a significant decrease in 1-year mortality the last 35 years, whereas other studies on temporal trends did not [6, 7].

The Swedish Hip Arthroplasty Registry (SHAR) provides a unique opportunity to study mortality on a large group of patients. Hip arthroplasty is considered the main treatment option for displaced femoral neck fractures for patients aged over 60 [8].

In this study, we aimed to identify the patients with the highest risk of dying after their fracture-related arthroplasty and to study the mortality, stratified by different patient, hospital and socioeconomic factors. Whilst previous studies have used the term absolute survival to study the increased risk, we intended to use techniques of relative survival analysis in an attempt to estimate and quantify the excess hazard introduced to our patients compared to the general population. We also studied if this excess hazard would either disappear in time, remain or continue to put the patient at higher risk of dying for several years after surgery.

## Methods

### Patients and methods

**Fig. 1:**
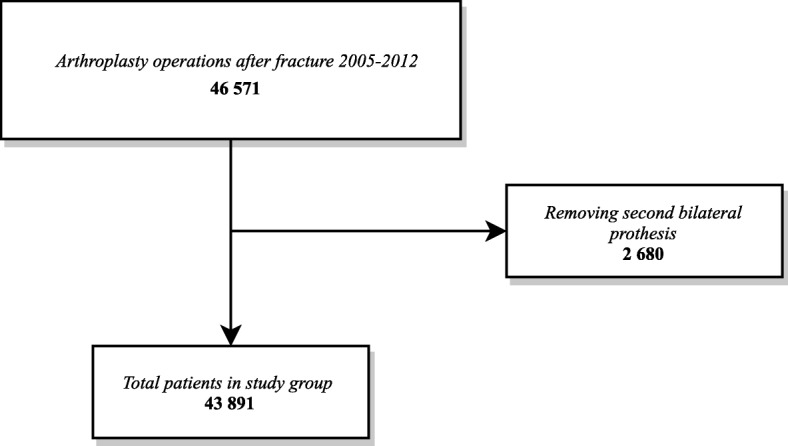
Patient flowchart

Since 2005 the SHAR registers hip fracture patients whom undergo both total and (or) hemiarthroplasty. We identified all patients operated between 2005 and 2012, and this patient group served as our study group. If a patient had sustained bilateral hip operations due to fractures, we only considered the first operation (Fig. 1). To adjust for confounding, accessible and relevant variables were collected from three registers. SHAR provided data about the patients’ age at operation, sex, prosthesis type (hemiarthroplasty or total hip arthroplasty), and hospital type (rural, county, university or private). The SHAR is a Swedish quality register and has a high completeness (98%) and full coverage (100%) [8]. Date of death is continuously updated in SHAR. The risk of leakage out of the register was considered, however only a handful of patients are known to have asked for their data to be removed [9]. Emigration rate in this group of frail elderly Swedes can be considered very low, and similar to the general population of the same age. Those few patients who emigrated were censored as they were dead [9]. Using the Swedish unique 10 digit personal identity number we linked the data from SHAR to Statistics Sweden and the National Patient Register (NPR) (National Board of Health and Welfare) [10]. Statistics Sweden provided data about the patients’ education and civil status, while the NPR delivered data about the patients’ health status encoded as ICD-10 codes. Swedish hospitals are obliged by law to report all primary and secondary ICD-10-codes to the NPR for every contact with the hospital. Only individual with entries in all three registers were included. Individual ICD-10-codes were combined into Elixhauser comorbidity indices (ECI) for each patient [11, 12]. The ECI is an unweighted comorbidity index representing the number of present comorbidities out of 31 possible. The ICD-10-codes were identified from hospital based healthcare in the year before the index hospital admission. Patents without recorded visits were classified as patients without known comorbidity (i.e. Elixhauser = 0).

### Statistical methods

We summarized continuous variables as means and standard deviations, categorical variables as counts and percentages. We used Student’s t-test and χ2-test for group comparisons. In this paper we used relative survival analysis [13, 14] The measure of interest was cumulative relative survival function r(t). We estimated the cumulative relative survival function as $\mathrm{r}\left(\mathrm{t}\right)=\frac{{\mathrm{S}}_{\mathrm{O}}\left(\mathrm{t}\right)}{{\mathrm{S}}_{\mathrm{P}}\left(\mathrm{t}\right)}$.

Where S_O_(t) denotes the observed survival and S_P_(t) the population or expected survival at time t. We extracted the Swedish population or expected survival S_P_(t) from publicly available mortality tables tabulated for sex and age maintained by the Human Life-Table Database [15] and Human Mortality Data Base [16].

The cumulative relative survival function r(t) can be any non-negative number. If r(t) = 1, then the observed survival of the studied group coincides with the expected or population survival. If r(t) > 1 then the observed survival of the studied group exceed the expected or population survival. If r(t) < 1 then the studied group experiences an excess mortality. We can partition the observed hazard for each individual λ_O_(t) into two additive components: λ_O_(t) = λ_P_(t) + λ_E_(t).

The age- and sex-specific baseline hazard is denoted by λ_P_(t). The difference between the baseline hazard and observed hazard, λ_E_(t) denotes the excess hazard of the study group.

Alongside with the cumulative relative survival, the excess hazard and the cumulative incidence curve served as the main methods of analysis. Additionally, we attempted to model the effect of clinical and patient related covariates with the help of multivariable Cox Proportional Hazards Model in Transformed Time [17]. We tested the assumption of proportionality with graphical examination and Brownian bridges [18]. We observed violation of the proportionality assumption for prosthesis type, age and ECI. We modelled these variables by the introduction of a step function that split the data in two epochs up to 14 months (420 days) and the subsequent period until end of study period. The multivariable regression analysis included interaction term between the aforementioned variables and the step function for time.

## Results

In the SHAR a total of 43,891 patients with hip arthroplasty surgery due to hip fracture were identified between 2005 and 2012(Fig. 1.) There was a female predominance (70%) and the average age was 79 years for the survival group and 84 for the deceased group (Table 1).

**Table 1:** Baseline demographic information for the study population stratified for survival status

	Alive	Dead	Total	*P*-value
Sample Size	22,575	21,316	43,891	
Sex: Female (%)	16,857 (74.7)	13,988 (65.6)	30,845	< 0.001
Age (mean & sd)	79.33 (9.17)	83.85 (7.77)		< 0.001
Hospital type (%)				< 0.001
University	5677 (25.1)	5916 (27.8)	11,593	
County	12,089 (53.6)	11,299 (53.0)	23,388	
Rural	3905 (17.3)	3351 (15.7)	7256	
Private	904 (4.0)	750 (3.5)	1654	
Prosthesis: Total hip arthoplasty (%)	7887 (34.9)	2690 (12.6)	10,577	< 0.001
Education (%)				< 0.001
Low	12,309 (54.5)	12,695 (59.6)	25,004	
Middle	6968 (30.9)	5740 (26.9)	12,708	
High	2793 (12.4)	1914 (9.0)	4707	
Missing	505 (2.2)	967 (4.5)	1472	
Civil status (%)				< 0.001
Couple	7971 (35.3)	6269 (29.4)	14,240	
Single	5489 (24.3)	4417 (20.7)	9906	
Widow	9071 (40.2)	10,620 (49.8)	19,691	
Missing	44 (0.2)	10 (0.0)	54	
Elixhauser index (mean & sd)	1.13 (1.35)	1.27 (1.53)		< 0.001
Elixhauser stratified (%)				< 0.001
0	9930 (44.0)	9534 (44.7)	19,464	
1	5448 (24.1)	4087 (19.2)	9535	
2	3754 (16.6)	3467 (16.3)	7221	
3+	3443 (15.3)	4228 (19.8)	7671	

**Fig. 2:**
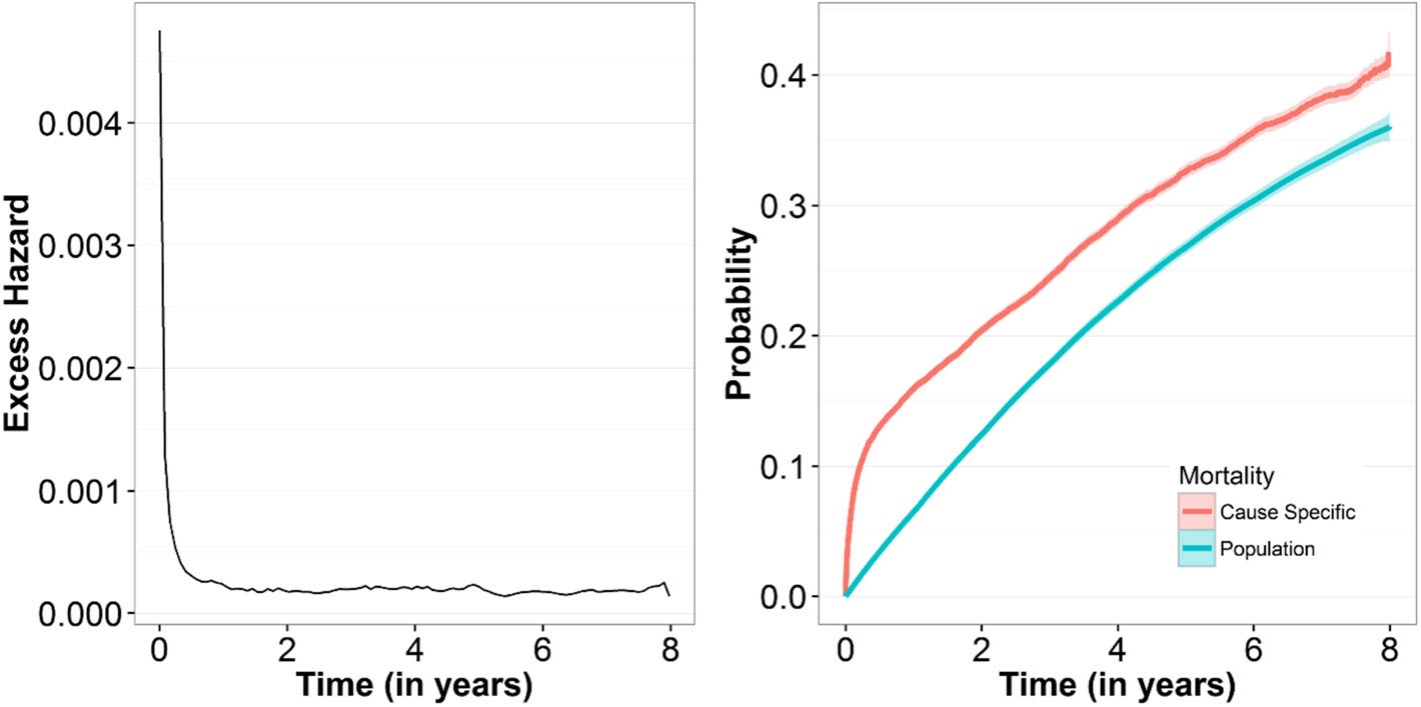
Excess hazard and crude mortality curves for 43,891 Swedish hip fracture patients compared with the mortality rates of the Swedish general population

As illustrated in Fig. 2 the baseline excess hazard of dying was elevated the first half year after the operation, thereafter the excess hazard decreased. However, it remained non-negligible trough the 8 years follow up period.

### Age and sex

**Fig. 3:**
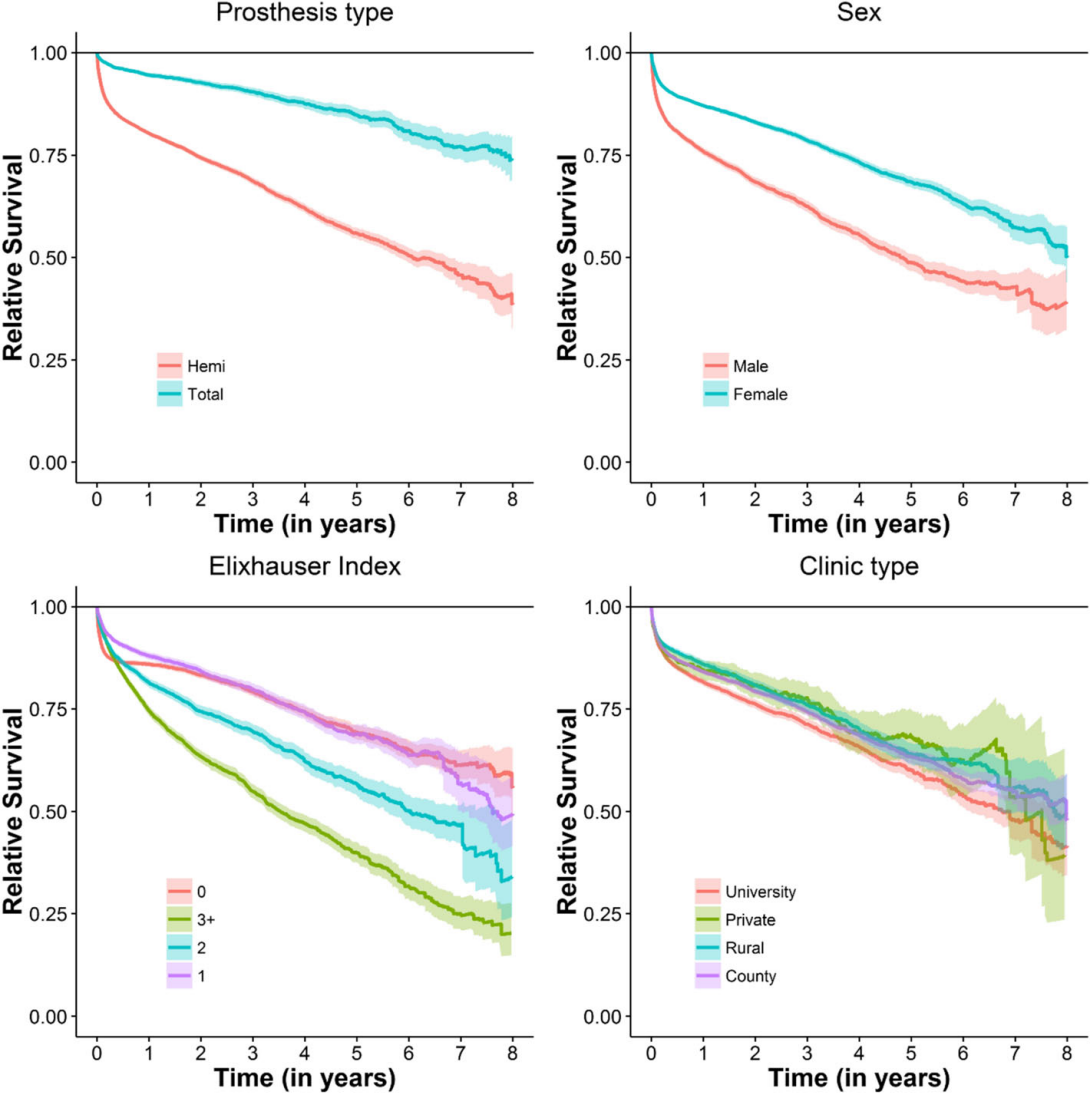
Relative survival curves of 43,891 Swedish hip fracture patients stratified in patient and hospital related factors. If the survival curves deviates the horizontal reference line then the survival of the studied stratum differs significantly from the general population

The mortality rate of males was higher, and both sexes had survival rates inferior than their peers from the general population (Fig. 3). Younger age, analysed as a continuous variable, led to an increased relative risk of dying, being more pronounced in the later period.

### Elixhauser comorbidity index

In general, the higher the ECI the lower the survival was. But the group with a ECI of 1 had better survival during the first 420 days, compared with those with ECI of 0 (Fig. 3). Over time the negative effect of the increasing ECI on survival became more obvious.

### Implant and hospital type

Patients with total hip arthroplasty had a better survival than patients with hemi-arthroplasty. Of the hospital types considered university hospitals had lower survival rate during the first 6 years, whilst patients operated at private, rural and county hospitals had similar survival rates. Over time, the impact of implant-type decreased (Fig. 3).

### Socioeconomics

**Fig. 4:**
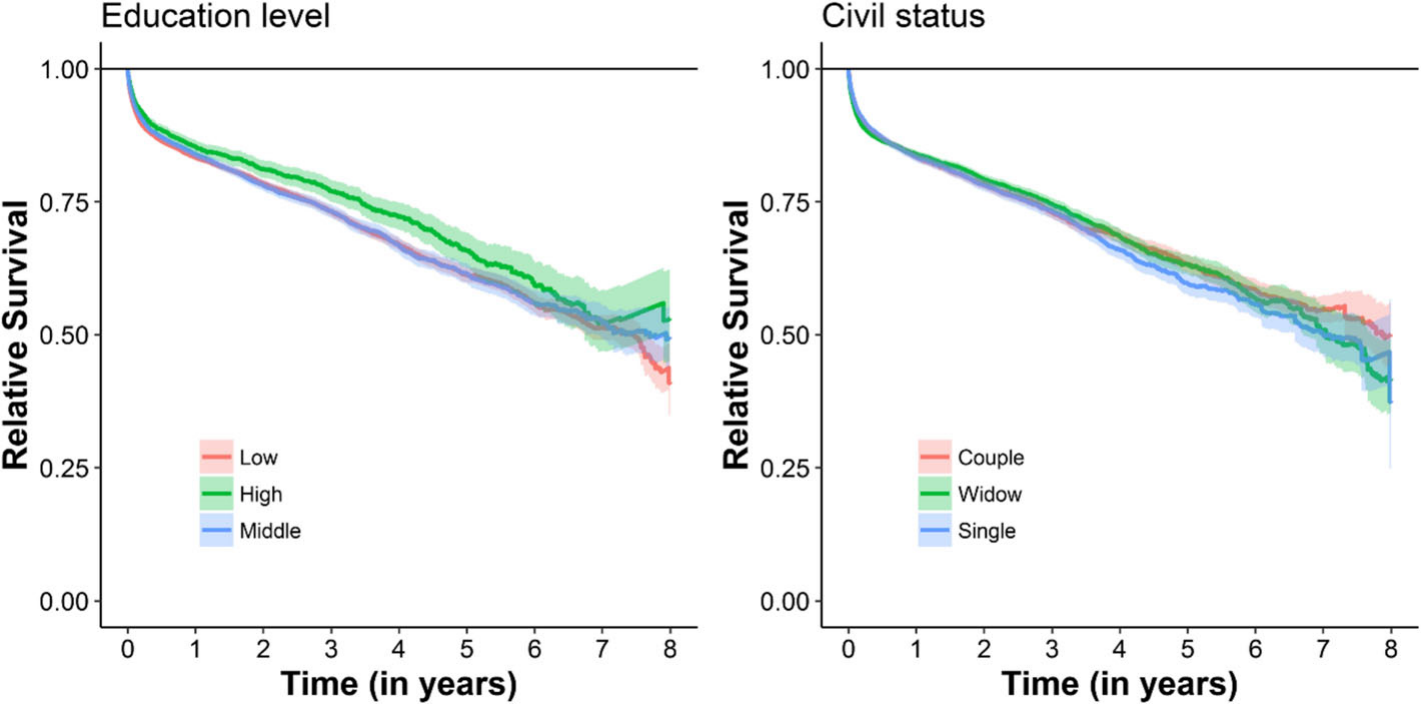
Relative survival curves of 43,891 Swedish hip fracture patients stratified in Education level and Civil status. If the survival curves deviates the horizontal reference line then the survival of the studied stratum differs significantly form from the general population

The separation of the survival rates for the different socioeconomic status was significant for education. Patients who completed higher levels of education had better survival that patients with low or middle education levels. (See Fig. 4) The later two categories had similar survival rates during the whole follow up period. Being married compared to being single and having higher achieved levels of education lowered the risk of death, however being a widow did not (Table 2). Multivariable relative survival regression corroborated most of the results of the univariate analysis.

**Table 2:** Multivariable relative survival regression analysis of survival of Swedish hip fracture patients

	HR	95% CI
Sex		
Female	*Ref*	
Male 0–420 days	2.48	2.37; 2.59
Male > 420 days	2.32	2.11; 2.55
Age		
0–420 days	0.94	0.94; 0.94
> 420 days	0.91	0.90; 0.92
Elixhauser 0–420 days		
0	*Ref*	
1	0.92	0.86; 0.97
2	1.23	1.16; 1.30
+ 3	1.47	1.39; 1.55
Elixhauser > 420 days		
0	*Ref*	
1	1.09	0.96; 1.24
2	1.30	1.15; 1.49
+ 3	1.75	1.55; 1.97
Year of operation		
0–420 days	1.00	0.99; 1.01
> 420 days	0.99	0.99; 1.01
Prosthesis		
Hemi	*Ref*	
Total 0–420 days	0.38	0.35; 0.41
Total > 420 days	0.54	0.46; 0.64
Hospital		
University	*Ref*	
County	0.95	0.92; 0.98
Rural	0.93	0.89; 0.97
Private	0.89	0.82; 0.95
Education		
Low	*Ref*	
Middle	0.99	0.96; 1.02
High	0.91	0.87; 0.95
Civil status		
Couple	*Ref*	
Single	1.08	1.03; 1.12
Widow	1.03	0.99; 1.06

## Discussion

### Main findings

Swedish hip fracture patients whom undergo hip arthroplasty had a high excess hazard of dying, up to 100 days after the operation compared with individuals in the general population of the same age and sex. This excess hazard diminished with time but never subsided to negligible levels. The mortality rate of hip fracture patients remained higher than the mortality rate of the general population during the whole 8 years’ follow-up period.

Earlier papers have related the long-term mortality among hip fracture patients to the mortality rates in the general population [3, 4, 6, 19]. These studies highlighted an increased mortality among hip fracture patients compared with the general population using age and sex standardization. We used relative survival rate and multivariable modelling and could therefore relate every patient to the survival rate of members of the general population of the same age and sex.

The excess early mortality can possibly be explained by the comorbidities and frailty of hip fracture patients, aggravated by the injury and the surgery. The relationship between fracture and increased risk of death up to 8 years post-surgery, is more difficult to disentangle. Apart from the chronic disease status in this patient population, previous research has suggested that pain and fear of falling [20] is contributing to a decrease in activity and exercise and a decrease in walking distance [21], which in turn potentially contributes to a loss of independence and depression [22]. In order to improve outcomes following hip fracture surgery, it is imperative to identify modifiable factors that could possibly aid survival and function [23]. There have been promising results where an improved and close cooperation between orthopaedic surgeons and geriatricians have led to a reduction in mortality [24] aided by systematic and sustained quality improvement efforts [25, 26].

### Age

Advanced age is associated with an increase in mortality after hip fracture however, this may be due to the higher general mortality rates that comes with aging [27, 28], as we by relative survival analysis observed that age was positively associated with the relative survival. Patients who suffer a hip fracture in their younger years lost more of their expected life span than patients who had their fracture in more advanced age. This effect increased with the follow-up time. This finding suggests that especially in the younger hip fracture population, there could be a greater benefit of an improved collaboration between healthcare professionals within the hospital setting, general practitioners and the municipal social care.

### Sex

The excess mortality of males was higher than for females. This was more evident in the beginning of the study period. Subsequently the excess sex-related differences in survival following hip fracture surgery follow a similar trends of the sex-related difference in survival of the general population. That men have a higher risk of dying after hip fracture is well known from the literature, and was shown also in an recent study [19]. It can be debated if this is induced by sex as an isolated variable, or if being male is a surrogate variable for comorbidity, unhealthy lifestyle or other confounders adding to their risk of both hip fracture and death.

### Elixhauser comorbidity index

Generally patient comorbidity had a significant adverse effect on survival [28], even though moderate comorbidity may not be associated with an excess one-year-mortality [27–31]. The hazard rates increased with increased score of comorbidity, as measured by the ECI [32]. This effect became stronger with time. Interestingly, in the early period patients with ECI = 1 have higher survival rate than patients with ECI = 0, while after 420 days no such difference was seen. An attempted explanation could be that patients with known comorbidities are receiving increased medical attention prior to the surgery, improving their health status. The group of patients with an ECI = 0 may consist of two types of patients; really healthy individuals and those who are not, but never sought medical attention and as such have undiagnosed and/or untreated comorbidity. Assumingly, in the hip fracture population there are a number of patients neglecting their health. These patients may present with an unstable medical condition at the time of their hip fracture, and subsequently have a higher risk of dying. We believe our results could question the validity of comorbidity indices based on administrative patient registers. Besides sick individuals not being known to the healthcare, there is a possibility of misdiagnosis bias due to incorrect coding [33].

### Hospital type

We found lower relative survival of patients treated at university hospitals. Similar results has been described in a Danish study [34], whilst a Canadian study found an improved survival in teaching hospitals compared to community hospitals [35]. Finally, a study from the USA concluded that hospital volume did not predict mortality, even though hospitals with high caseload had fewer complications [36]. Swedish university hospitals typically serve an urban population, so when comparing different hospitals, we also compare different populations. Based on the available evidence one could conclude that both hospital level and volume are blunt variables confounded by case-mix, level of competence of the staff and an unclear definition of what high volume is, making comparisons between different healthcare systems difficult.

### Year of surgery

The year of surgery was not associated with any excess risk of mortality, indicating that during our study period the rate between hip fracture patients survival and the survival of the general population did not change. This finding is in agreement with the findings of Klop et al. [37]. In a systematic review on randomized clinical trials, Mundi et al. found similar mortality in hip fracture patients over time [6].

### Implant type

Patients operated with total hip arthroplasty had lower mortality than those with hemi-arthroplasty. This is most probably a reflection of selection bias as choice of implant is based on patient frailty, age and activity level.

### Socioeconomics

Solitary life style [38] and social deprivation [39] have been previously associated with increased mortality after hip fracture. In our study we found similar results, where being non-married brought on a slight increase in mortality. However, in our study being widowed had no adverse effect on mortality.

### Strengths and limitations of the study

Other factors may influence mortality. Solbakken and collaborators [40] concluded that self-perceived health, smoking, and BMI predict mortality of hip fracture patients. Vosoughi and collaborators [41] reached similar conclusions regarding smoking and BMI and noted improved nutrition as modifiable factors. Hence, as our register data is limited in terms of such variables, our results could not be adjusted for those and might have to be interpreted with caution due to varying degree of validity [42].

The SHAR has an excellent completeness of over 98% [8], in combination with the large patient cohort are the main strengths of the present study. The additional access to socioeconomic and comorbidity data from governmental administrative registers further add to the strength [10]. Additionally, the use of relative survival analysis has an overarching advantage that, through a single measure, it indicates the degree to which a study sample corresponds to the general population with respect to health status and survival [43].

### Perspective

Orthopaedic healthcare of today focuses on a timely and effective treatment of hip fracture patients. Post-operative care can also be of a fast-track nature, both reflecting new scientific knowledge and health economic challenges. Concerns have been raised whether a shorter length-of-stay is associated with an increase in mortality [44]. Our findings may assist the clinician to identify patients at risk of excess mortality. The patient without prior medical records or diagnoses can actually be more challenging than expected. A younger patient will suffer a risk of a greater expected life span loss than the more elderly patient and this should not be neglected. The same goes for singles and/or males. The hip fracture patient in general carries a highly increased risk of dying during several months after fracture, compared to the general population, and further closer follow-up care have to be provided after discharge from the hospital.

## Conclusions

Hip fracture patients, in particular males, have a considerable excess hazard of dying compared with the general population. Younger patients have a greater loss of expected life span than patients who suffer hip fracture in their more advanced ages. Existing comorbidities, lower education and solitary life style all have an adverse effect on survival. The survival rates of hip fracture patients did not improve during a study period of 7 years.

## Abbreviations

ECI: Elixhauser comorbidity index
SHAR: The Swedish Hip Arthroplasty Registry
